# New Method of Tying Divided Arteries

**Published:** 1837

**Authors:** Daniel Stahl

**Affiliations:** Vincennes, Indiana


ART. V.—REMARKS ON A NEW METHOD OF TYING DIVIDED ARTERIES. By Daniel Stahl, M. D., of Vincennes, Indiana.

In the last number of the Western Journal of the Medical and Physical Sciences* is an article by Dr. Portal, "on the

---

*p. 421

ligature of arteries in case of aneurism," which, although not a new invention (see Chelius' Handbuch der Chirurgie, third edition, p. 151) *may be* a useful improvement in that branch of operative surgery. I say "*may be*," because the experiments and observations of Jones make the success of Dr. Portal's method rather doubtful. But even admitting that simply bringing the internal coats of the vessels in contact in cases of aneurism will prevent secondary hemorrhage, the question still arises whether the method is applicable in cases of divided arteries? As this question has not yet been decided, I beg leave to present the reader with the following extract from a letter which I received in 1835 from my friend, Dr. B. Stilling, of Capel, in Germany. He writes thus: "Since you left Germany, I have discovered a method for closing large arteries without ligature or torsion and without danger of supervening suppuration. It is described in my work on the "Die Gefiissdurchsechlingung," published in two volumes at Marbury, in 1834, and the following is an outline of it.

"The end of the divided artery, embraced by a pair of forceps, is to be drawn out and flattened. A longitudinal cut or slit is then to be made through the coats of the vessel, a little to one side of it; and through this opening the forceps are next to be carried, and after being made to sieze the divided extremity of the artery, they are withdrawn. By this manœuvre the vessel, is completely closed, and will remain so, provided the slit at the side of the flattened end is as long as the *tumor*, and from half an inch to an inch distant from the margin. To prevent bleeding during the operation, it is necessary that the artery should be compressed a little above the slit by means of a pair of forceps.

" I have performed this operation," adds the doctor, "once upon a man, and one hundred and twenty-three times upon animals, and always with the greatest success. Many of the experiments were witnessed by Professors Ullmann, Bunger and Hensinger, of Marburg."

Dr. Stilling is a young man of very superior talents, and is well known in Germany by his ingenious experiments in operative surgery and by his writings. His statements can be relied upon; and as his work seems to be still unknown on this side of the Atlantic, I considered it a duty which I owe to the profession to communicate the above remarks.

*March*, 1837.

---

### Editorial Remarks.

We regret that it has not been in our power to publish the drawings, which the author of the above article has sent us as illustrative of the different steps of Dr. Stilling's new and ingenious operation. The printing of the Journal was so far advanced when the paper reached us as to render any thing of the kind impracticable. Will not some of our country friends take up this subject, and experiment upon it? The operation is certainly highly ingenious, and worthy of the deepest consideration of the profession.

